# Crosstalk between periodontitis and cardiovascular risk

**DOI:** 10.3389/fimmu.2024.1469077

**Published:** 2024-12-09

**Authors:** Ulrike Schulze-Späte, Ludwig Wurschi, Emiel P. C. van der Vorst, Frank Hölzle, Rogerio B. Craveiro, Michael Wolf, Heidi Noels

**Affiliations:** ^1^ Section of Geriodontics, Department of Conservative Dentistry and Periodontics, University Hospital Jena, Jena, Germany; ^2^ Institute for Molecular Cardiovascular Research (IMCAR), Uniklinik RWTH Aachen, RWTH Aachen University, Aachen, Germany; ^3^ Aachen-Maastricht Institute for Cardiorenal Research (AMICARE), Uniklinik RWTH Aachen, RWTH Aachen University, Aachen, Germany; ^4^ Interdisciplinary Center for Clinical Research (IZKF), RWTH Aachen University, Aachen, Germany; ^5^ Institute for Cardiovascular Prevention (IPEK), Ludwig-Maximilians-University Munich, Munich, Germany; ^6^ Department of Oral and Maxillofacial Surgery, School of Medicine, Uniklinik RWTH Aachen, Aachen, Germany; ^7^ Department of Orthodontics, Dental Clinic, Uniklinik RWTH Aachen, Aachen, Germany; ^8^ Biochemistry Department, Cardiovascular Research Institute Maastricht (CARIM), Maastricht University, Maastricht, Netherlands

**Keywords:** periodontitis, cardiovascular disease, innate immunity, inflammation, dysbiosis

## Abstract

Recent demographic developments resulted in an aged society with a rising disease burden of systemic and non-communicable diseases (NCDs). In cardiovascular disease (CVD), a NCD with high morbidity and mortality, recent preventive strategies include the investigation of comorbidities to reduce its significant economic burden. Periodontal disease, an oral bacterial-induced inflammatory disease of tooth-supporting tissue, is regulated in its prevalence and severity by the individual host response to a dysbiotic oral microbiota. Clinically, both NCDs are highly associated; however, shared risk factors such as smoking, obesity, type II diabetes mellitus and chronic stress represent only an insufficient explanation for the multifaceted interactions of both disease entities. Specifically, the crosstalk between both diseases is not yet fully understood. This review summarizes current knowledge on the clinical association of periodontitis and CVD, and elaborates on how periodontitis-induced pathophysiological mechanisms in patients may contribute to increased cardiovascular risk with focus on atherosclerosis. Clinical implications as well as current and future therapy considerations are discussed. Overall, this review supports novel scientific endeavors aiming at improving the quality of life with a comprehensive and integrated approach to improve well-being of the aging populations worldwide.

## Introduction

Within the ageing global population, the disease burden of systemic and non-communicable diseases (NCDs) is increasing (1, 2). Cardiovascular disease (CVD) represents most cases in this context and it is globally the leading cause of death, being responsible for 32% of all global deaths in 2019 (3). Hence, defining effective ways for prevention and treatment of CVD is of great importance. This often involves detailed analysis of local and systemic tissue pathogenesis in association with the disease. Among the diseases associated with CVD, periodontitis (PD) is particularly common. PD is a chronic inflammatory disease affecting the tooth-supporting tissues (4) and severe PD is present in approximately 19% of the global population older than 15 years, representing more than 1 billion cases, which makes it one of the most common NCDs (5). By itself, PD has been associated with all-cause mortality as well as with mortality following cardiovascular events (6). By researching influencing factors, the noticeable association of both diseases has been continually established and will be described in detail in this review. Specific focus will be on the missing links of this multifaceted crosstalk that potentially could affect outcome, address therapeutic resistance and open novel avenues for preventive measures in the aging population.

## Periodontitis: pathophysiology

### General pathophysiology

**Figure 1:**
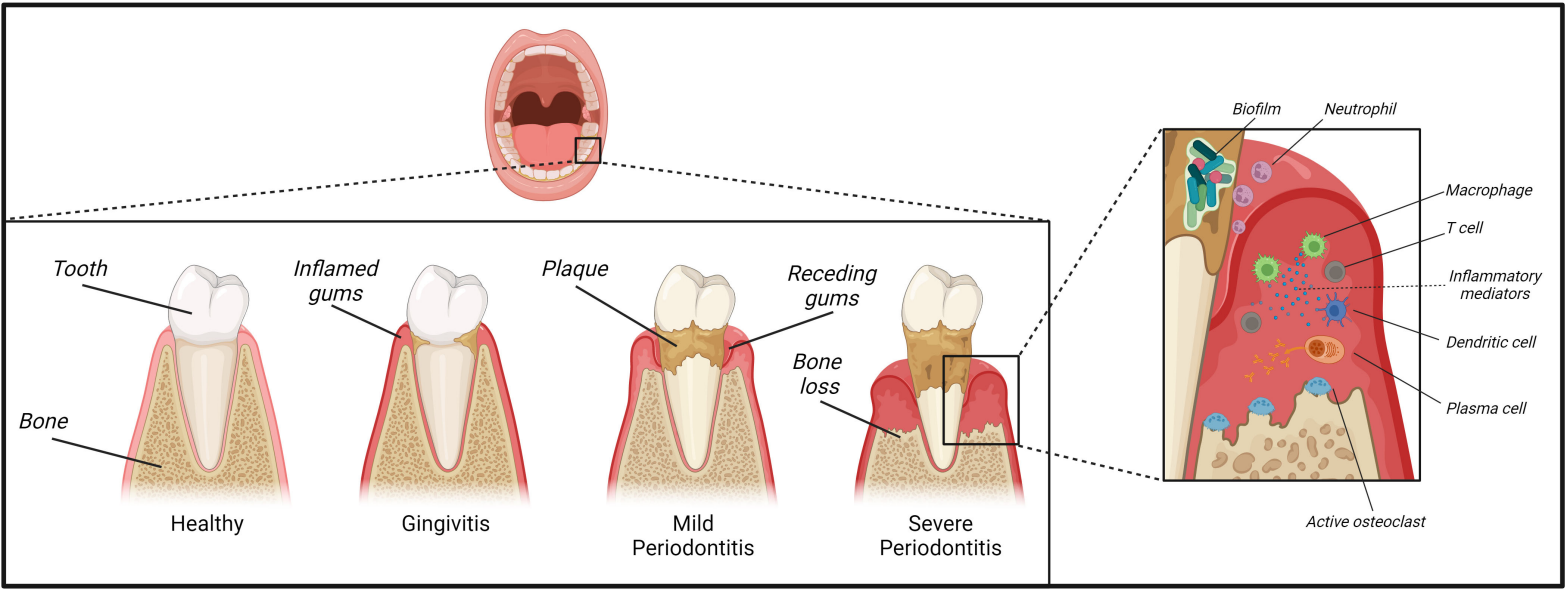
Development and pathophysiology of periodontitis. Plaque deposited on our teeth provides a substrate for a variety of bacterial species. A lack in oral hygiene and insufficient plaque removal from the tooth surfaces and their surrounding junctional gingival tissue can trigger the development of gingivitis. Inflammation can then further progress towards the development of mild to severe periodontitis paralleled by establishment of a dysbiotic biofilm of inflammophilic bacteria. Initially, the periodontal tissue is infiltrated by neutrophils, T-lymphocytes and mononuclear cells such as macrophages, followed by mainly plasma cells in a later stage. These plaques can potentially expand into a destructive advanced lesion inducing loss of periodontal ligament and alveolar bone and thus causing the characteristic clinical symptoms of periodontitis.

PD is a disease of the tissues attaching the teeth, where dental plaque accumulation and the propagation of bacterial key pathogens within an oral dysbiosis provoke an escalated inflammatory host response and impaired resolution of inflammation, leading to periodontal tissue damage and subsequent loss of alveolar bone and teeth (7–10) (**Figure 1**). It is part of the spectrum classifying inflammatory diseases of the periodontium categorized in a collaborative work by the American Academy of Periodontology (AAP) and European Federation of Periodontology (EFP) (11, 12). Its characteristic clinical symptoms comprise gingival bleeding, attachment loss, dental hypermobility, halitosis and subsequent loss of alveolar bone and teeth (11). Besides these local symptoms, PD leads to systemic consequences (10) and its connection to conditions such as type II diabetes mellitus (T2DM), rheumatoid arthritis, obesity and CVD has been of rising interest in the past decades CVD (13–15). Overall, PD has become a global health problem with an economic burden due to the global demographic development with a growth in the aging population and subsequent increasing prevalence in the elderly (16, 17).

### Local pathophysiology

The tissue destructive process of PD is associated with the establishment of a plaque-induced dysbiosis (18), subgingival microbial communities, and the presence of responsive immuno-inflammatory infiltrates (14). Opposite to PD as a tissue destructive disease of tooth-supporting tissue, gingivitis is a reversible inflammatory condition of the marginal gingival tissue attachment, which results from plaque accumulation or manifestation of an underlying systemic disease (19). In gingivitis, the inflammatory response to bacterial plaque is contained within epithelium and connective tissue without affecting deeper bony compartments. The prevalent aetiological path is a lack in oral hygiene and insufficient plaque removal from the tooth surfaces and their surrounding junctional gingival tissue (20). This plaque provides a substrate for a variety of bacterial species (21, 22) that are abundant in a planktonic form within the saliva to colonize these dental surfaces and form a matrix that provides protection against elution, phagocytosis, mechanical stress and antimicrobial salivary factors (23). The oral cavity is home to approximately 700 predominant bacterial taxa that only partly have been cultivated *in vitro* (24).

Gingivitis can, however, be both the precursor, risk factor and accompanying symptom of a later PD (14). Within the maturing biofilm, potentially pathogenic species establish themselves and elucidate a destructive inflammatory response by the immune system. Bacterial presence is required for PD pathogenesis in the susceptible host, however, predominantly an ineffective and dysregulated host response to the microbial challenge drives periodontal tissue damage (24). Non-resolving inflammation and the selective support of dysbiotic inflammophilic communities result in a vicious cycle that is the hallmark of chronic PD and its associated tissue loss (14). In contrast, a tissue-protective immune response fosters balanced host-microbe interactions resulting in tissue homeostasis within a healthy periodontium. Compared to gingivitis or PD, lower numbers of neutrophils as the early responder cells are present together with a small population of γδ T cells and innate lymphoid cells (25, 26).

To describe the developmental process of PD and its associated cellular cascades, Page and Schroeder first established a progressive model, which subsequent studies used to expand on (27, 28). Thereby, PD correlates with an increasingly complex cellular infiltrate- with neutrophils dominating an initial lesion characterized by beginning degradation of perivascular collagen and an exudative vasculitis. Neutrophils arrive from the circulation to the afflicted site and complex molecular interactions with the endothelium involve distinct chemokine- and selectin-induced signaling and adhesive β2 integrins cascades (14). Furthermore, pro-inflammatory mediators such as arachidonic acid derivatives [e.g. Prostaglandins and leukotrienes (29)], and complement activation products, e.g. anaphylatoxins C3a and C5a, can influence neutrophil extravasation and drive pathogenesis of PD (30). Subsequently, inflammation progresses into an early lesion, where periodontal tissue is infiltrated by T-lymphocytes and antigen-presenting cells such as macrophages and dendritic cells (DCs). Ultimately, B and plasma cells dominate the periodontal infiltrate in a fully established lesion, although bone loss does not commence yet (14). This established lesion may remain stable for a prolonged period, but it potentially expands into a destructive advanced lesion inducing loss of periodontal ligament and alveolar bone - thus causing the characteristic clinical symptoms of PD (11, 31).

### The oral microbiome

Previous analysis of the oral microbiome in PD patients involved systematization of the bacterial communities based on adaptation to aerobic versus anaerobic conditions and bacterial virulence factors during different stages of disease progression (32). With mineralization of the plaque and its progression into the sulcus, the inflammatory focus moves into the deepening gingival crevice, which corresponds to establishment of an increasingly anaerobic milieu. Thus, presence of Streptococcus species such as *S. mitis*, *S. oralis*, *S. intermedius* and *S. gordonii*, followed by *Actinomyces naeslundii* and *odontolyticus, Veillonella parvula* and *Fusobacterium nucleatum* and subsequently *Porphyromonas gingivalis* (*P. gingivalis*), *Treponema denticola* and *Tannerella forsythia* together with *Aggregatibacter actinomycetemcomitans* (*A. actinomycetemcomitans*) were described in decreasing order. However, recent findings focused on the identification of specific key pathogens (e.g. *P. gingivalis*) within the polymicrobial community and the establishment of dysbiosis, an alteration in abundance and influence of individual species within the biofilm (14). Keystone pathogens, assisted by accessory pathogens in the provision of nutrients and colonization, can initially escape host immunity, which allows them to establish a dysbiotic microbiota that also includes commensal-turned pathobionts. Ultimately, polymicrobial dysbiotic communities over-activate the inflammatory host response and periodontal tissue loss progresses.

### From local to systemic inflammation

Additionally, generalized spread of causative bacteria, bacterial products and mediators of non-resolving inflammatory pathways could compromise systemic tissue homeostasis. Hence, locally produced pro-inflammatory cytokines such as tumor-necrosis factor alpha (TNFα), interleukins (IL)-1 or IL-6 and prostaglandin E_2_ could move via systemic circulation to distant organs (33) and contribute to cellular disbalance far away from the side of infection. In general, this supports PD`s crosstalk and association with other chronic systemic diseases such as diabetes, obesity, cardiovascular and musculoskeletal diseases (7, 34). The immune reaction shows great individual variability (35) and it is influenced by multiple factors such as genetic, epigenetic, environmental (e.g. smoking, stress, diet) factors, aging and underlying diseases such as T2DM (36), Papillon-Lefèvre syndrome (37), osteoporosis, obesity or specifically atherosclerosis and CVD (13–15).

### Cellular and molecular players of the involved immune response

Certain cellular signaling cascades play a special role in the immune reaction. At first, a reaction of the innate immune system is occurring, where bacteria are identified by certain pathogen-associated molecular patterns via pattern-recognition receptors such as toll-like receptors (TLR) of DCs or macrophages. These antigen-presenting cells release a variety of pro-inflammatory cytokines and chemokines and activate surrounding periodontal ligament fibroblasts, thrombocytes and polymorphonuclear leukocytes. The developing cytokine cascade, comprising factors like TNF-α, IL-1β, IL-6, IL-8, IL-12, IL-17 or Prostaglandin E2, amplifies the immunological response (38). TNF-α and IL-1β cause a disinhibition of nuclear factor kappa B (NF-κB), a transcription factor promoting the transcription of pro-inflammatory genes. Subsequently this leads to the expression of matrix metalloproteinases like Collagenase 1 (MMP-1) by macrophages and fibroblasts, Collagenase 2 (MMP-8) by macrophages, macrophage elastase (MMP-12) by T-cells, neutrophils, macrophages and fibroblasts, Gelatinase A (MMP-2) by fibroblasts and Gelatinase B (MMP-9) by neutrophils (39). These MMPs are responsible for the degradation of the extracellular matrix when the resolution of inflammation fails and a chronic condition develops. The IL-17-stimulated expression of the receptor activator of NF-κB ligand (RANKL) by osteoblasts and periodontal ligament cells leads together with macrophage colony-stimulating factor to the activation, fusion and differentiation of osteoclast progenitor cells (40). RANKL is also produced by activated T and B lymphocytes in the inflamed periodontal tissue. Consequently, the balance of osteolysis and osteogenesis is displaced and results in alveolar bone resorption (41). Of note, neutrophils not only support acute but drive chronic inflammation processes by recruiting and stimulating Interleukin-17 release via Th17, CD4+ T helper cells (42). The inflammatory response is counteracted by the network activation of the inflammation-resolution response that is promoted by lipid-metabolic factors such as lipoxins, protectins, maresins, 4-hydroxydocosahexaenoic acid (4-HDHA), 17-HDHA or resolvin E1 (43, 44). For instance, the application of resolvin E1 has been shown to reduce C-reactive protein (CRP), intima-media ratio, leukocyte infiltration, formation of arteriosclerotic plaques, and periodontal inflammation in general (45). Furthermore, immunomodulatory interventions support direct involvement of additional lipid-metabolic factors such as fatty acids and associated ceramides (9), diacylglycerols, triglycerides and stress-reducing lipokines (1,2-dioleoyl-sn-glycero-3-phospho-(1’-myo-inositol) [PI(18:1/18:1)]) (46) in periodontal cellular cascades impacting tissue homeostasis and alveolar bone microarchitecture (9).

In summary, periodontitis involves a chronic oral microbial dysbiosis and overactivation of the host immune response, which ultimately result into a locally destructive lesion triggering a progressive loss of periodontal tissue as well as chronic systemic inflammation.

## Cardiovascular disease and atherosclerosis: pathophysiology

### General pathophysiology

**Figure 2:**
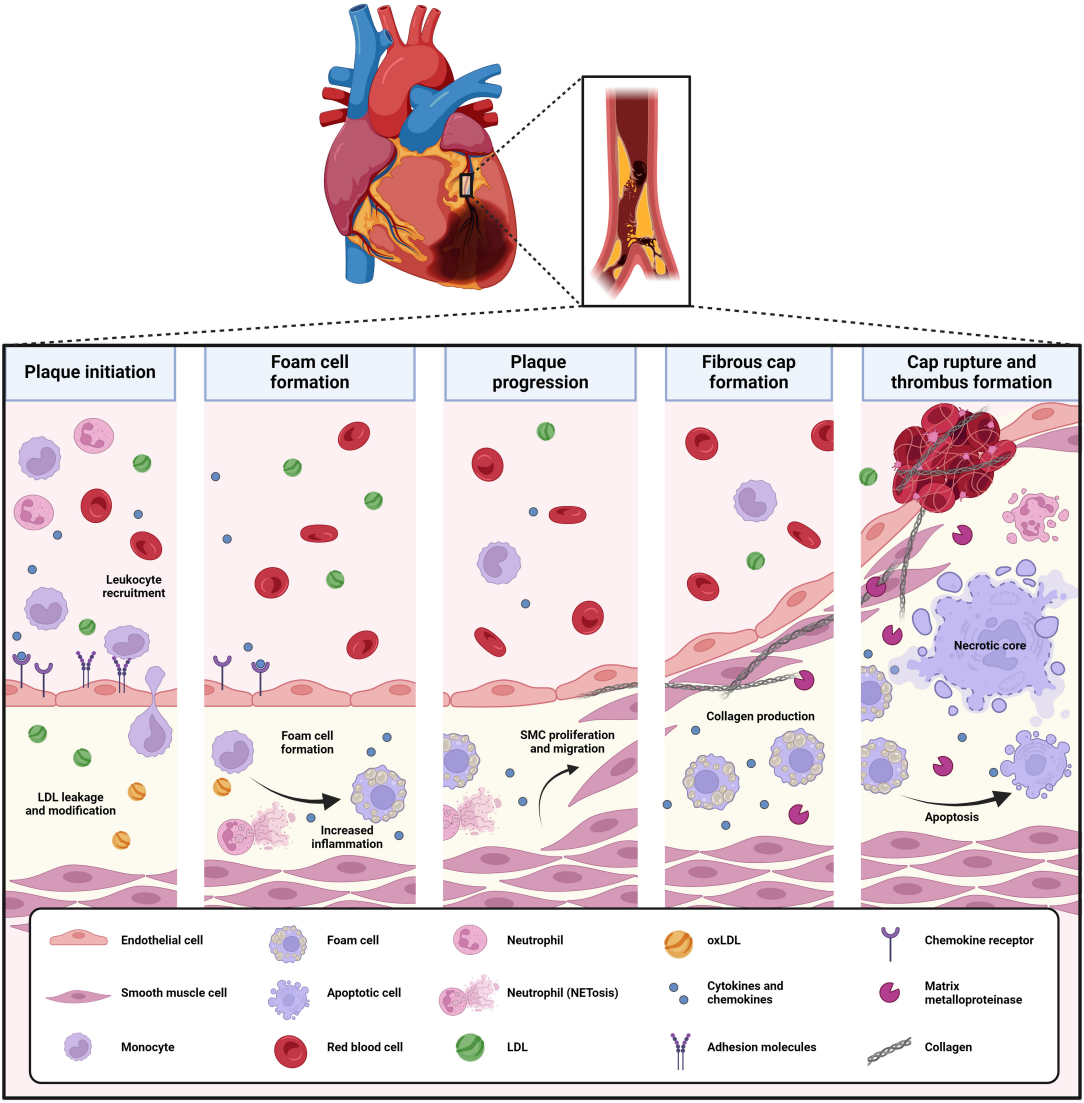
Development and pathophysiology of atherosclerosis. Atherosclerosis is a progressive disease driven by lipid deposition and inflammation into the arterial wall. Initial endothelial dysfunction triggers LDL leakage in the vessel, where it is oxidized to oxLDL. Also, it induces the recruitment and adhesion of leukocytes (neutrophils, monocytes, T-lymphocytes) and their migration into the vascular intima. Here, oxLDL drives monocyte-derived macrophages to lipid uptake and foam cell formation, triggering a pro-inflammatory profile and ultimately, lipid overload and cell death. Neutrophils elucidate inflammatory responses by secreting pro-inflammatory mediators as MPO, produce ROS and contribute to the recruitment and activation of monocytes/macrophages by secreting chemotactic granule proteins as well as the production of NETs. Vascular SMCs migrate from the vessel wall into the developing atherosclerotic lesion, where they form a protective fibrous cap. Upon plaque progression and increasing inflammation, the fibrous cap thins, which makes the atherosclerotic plaque prone to rupture and trigger thrombus formation. LDL, low-density lipoprotein; MPO, myeloperoxidase; NETs, neutrophil extracellular traps; oxLDL, oxidized LDL; ROS, reactive oxygen species; SMCs, smooth muscle cells.

Atherosclerosis is an important underlying pathology of coronary artery syndrome and refers to the development and progression of lipid-rich, inflammatory lesions in the arterial wall (**Figure 2**). By severely reducing the vessel lumen or by inducing thrombosis through atherosclerotic plaque rupture or erosion, atherosclerosis can ultimately trigger acute coronary syndrome, myocardial infarction and sudden cardiac death. Mechanistically, atherosclerosis is driven by a combination of hyperlipidemia [with high levels of low-density lipoprotein-cholesterol (LDL-C) and triglycerides but reduced levels of high-density lipoprotein-cholesterol (HDL-C)] and systemic inflammation. Triggered by initial endothelial dysfunction - characterized by endothelial inflammation and increased endothelial permeability -, LDL and inflammatory leukocytes gradually accumulate into the subintimal space of the arterial wall, where they drive vascular inflammation (47). The pro-inflammatory milieu in the vasculature triggers the oxidation of LDL to oxidized LDL (oxLDL), which drives macrophage foam cell formation by oxLDL uptake and pro-inflammatory responses in both macrophages and endothelial cells (47). Also, polymorphonuclear granulocytes or neutrophils infiltrate the vascular wall, where they elucidate inflammatory responses by secreting pro-inflammatory mediators as myeloperoxidase (MPO), produce reactive oxygen species (ROS) and contribute to the recruitment and activation of monocytes/macrophages by secreting chemotactic granule proteins as well as the production of neutrophil extracellular traps (NETs) (48). Lipid overload triggers macrophage death and the formation of necrotic cores with high-inflammatory potential. Smooth muscle cells (SMCs) can migrate from the media into the atherosclerotic lesion to form a protective fibrous cap. However, as for macrophages, lipid loading can trigger a phenotyping switching of SMCs, which increases their inflammatory potential and can induce SMC apoptosis and necrosis (48).

Beyond neutrophils and monocytes/macrophages, also DCs and T-lymphocytes influence atherosclerosis. Among T-cells, Th1 and Th17 cells drive atheroprogression, whereas regulatory T-cells (Tregs) are atheroprotective (47, 49). Also, DCs are found in atherosclerotic lesions, where they contribute to pro-inflammatory cytokine production and Th1 cell polarization (47).

Upon atherosclerotic plaque progression, the production of collagenases and other enzymes degrading extracellular matrix proteins - for example neutrophil elastase produced by neutrophils - triggers the degradation of the fibrous cap, making the plaque increasingly prone to rupture. In such case, the content of the atherosclerotic lesion is exposed to the blood stream, which triggers instant thrombosis (47). On the other hand, plaque disruption with subsequent thrombosis can also be caused by superficial erosion of atherosclerotic lesions, which - relatively to plaque rupture - is increasing in importance due to the improved success of controlling LDL levels in CVD patients (50). Clinical data suggest that plaque erosion may account for around one third of acute coronary syndrome cases (51, 52). Mechanistically, plaque erosion is triggered by a disturbed blood flow triggering TLR2-mediated activation, death and desquamation of the endothelium, followed by neutrophil adhesion and NET formation (53). Plaque erosion occurs also in plaques that have a relatively low lipid-content and inflammatory profile (52).

### Endothelial dysfunction, systemic inflammation and innate immune activation as link between CVD and its comorbidities

All combined, this highlights the crucial contribution of endothelial dysfunction and systemic inflammation to atherosclerosis and CVD risk, and explains why comorbidities that impact on each of these processes, also enhance cardiovascular risk. This has for example been shown for chronic kidney disease (CKD), which elicits endothelial dysfunction and a chronic, low-grade systemic inflammation along with increased cardiovascular risk (54–57). Similarly, T2DM as important risk factor for CVD triggers endothelial dysfunction, systemic inflammation and oxidative stress (58) and also caloric intake and obesity induce an increased mobilization of monocytes (59, 60) and neutrophils (59) into the periphery, where these myeloid cells contribute to increased inflammatory and cardiovascular risk. In recent years, an increasing number of studies revealed that inflammatory triggers can prime hematopoietic stem and progenitor cells (HSPCs) in the bone marrow for adaptation towards increased myelopoiesis, a pathological mechanism that has now been recognized to crucially contribute to increased cardiovascular risk induced by cardiovascular risk factors like obesity, hyperlipidemia, chronic stress or sleep interruption, amongst others (61). For example, in obesity, adipose tissue-derived S100A8/A9 triggers IL-1β production by adipose tissue macrophages, which then signals to IL-1 receptor-expressing bone marrow myeloid progenitor cells to increase neutrophil and monocyte production (59). Chronic stress triggers sympathetic nervous system activation and noradrenaline production, which signals to bone marrow niche cells over the β3 adrenergic receptor to reduce CXCL12 production in the bone marrow and increase HSPC proliferation, resulting in a higher production of monocytes (monocytosis) and neutrophils (neutrophilia) (62). Of note, HSPCs can retain this capacity for increased myelopoiesis for a prolonged time even after resolution of the initial trigger. This process - referred to as ‘trained myelopoiesis’ - has now been acknowledged as a very important aspect of maladaptive innate immune adaptation. Mechanistically, it can be induced by epigenetic changes in HSPC triggering bone marrow memory. For example, hyperlipidemia was shown to trigger long-lasting NLRP3-dependent transcriptional and epigenetic reprogramming of granulocyte-monocyte progenitor cells with increased responsiveness to inflammatory triggers (63).

In summary, hyperlipidemia, endothelial dysfunction, systemic inflammation and innate immune activation are important drivers of atherosclerosis.

## Clinical association of periodontitis and atherosclerosis

The association between cardiovascular and periodontal disease is supported by an increasing evidence reviewed by Sanz et al. in a consensus report based on a collaborative work of AAP and EFP (64). When PD is present, the risk to develop CVD is generally increased, being possibly raised by 25%, and the likelihood to suffer particularly from a myocardial infarction (MI) or to develop a first MI is elevated too (65–67).

Furthermore, edentulism as a severe symptom of chronic PD is associated with CVD as well (6, 68, 69), however other causes of edentulism besides PD exist. On the contrary, a comparatively lesser degree of tooth loss correlates favorably with systolic blood pressure, glomerular filtration rate, levels of blood glucose or LDL-C, waist circumference and high-sensitivity C-reactive protein (hs-CRP), risk factors and parameters of CVD and metabolic syndrome (70). The degree of severity, represented by the stage of PD, also associates with the risk for CVD (71) and older patients with CVD reversely show signs of periodontal infection (72). Furthermore, patients suffering heart failure, a pro-inflammatory syndrome with multi-organ involvement caused by the inability of the heart to meet the metabolic demands of the body, exhibit more severe periodontal disease associated with increased bone turnover markers when compared with control patients (73).

As a limitation of these clinical studies, the co-existence of periodontitis and CVD may be triggered by shared risk factors, such as smoking, obesity, T2DM and chronic stress. These factors influence the progression of both PD (14) and atherosclerotic CVD (74), and may thus be potential confounders in the association of both diseases.

## From shared risk factors to PD-induced pathophysiological effects contributing to atherosclerotic risk

**Figure 3:**
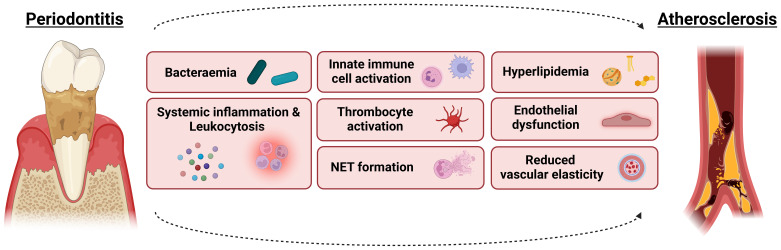
From shared risk factors to periodontitis-induced pathophysiological effects contributing to atherosclerotic risk. The co-existence of periodontitis and atherosclerosis may be triggered by shared risk factors such as smoking, obesity, T2DM and chronic stress. However, the pathogenesis of periodontitis could also by itself be responsible for the detected associations since the oral dysbiosis could influence cardiovascular health and existing atherosclerotic lesions directly and indirectly. Involved mechanisms include bacteraemia, systemic inflammation and leukocytosis, innate immune cell activation, hyperlipidemia, endothelial dysfunction and reduced vascular elasticity, thrombocyte activation as well as the formation of neutrophil extracellular traps (NETs). For more details, see text.

However, in addition to a simultaneous development due to shared risk factors, PD pathogenesis could also by itself be responsible for the detected associations since the oral dysbiosis could influence cardiovascular health and existing atherosclerotic lesions directly and indirectly, as discussed below and visualized in **Figure 3**.

### Bacteraemia

Periodontal tissues are intensely perfused and the hyperaemia in case of inflammation could cause both clinically visible bleeding on probing and bacteraemia, where microbes originating in the dental biofilm enter the bloodstream through vascular lesions. In patients with PD, nearly half of them showed bacteraemia after therapeutic periodontal treatment (75). In this way, periodontal bacteria can spread throughout the body, where they can promote inflammatory processes by disrupting immune surveillance mechanisms of the host, as discussed in detail elsewhere (4, 76, 77).

For example, the prominent parodontopathogens *A. actinomycetemcomitans* and *P. gingivalis* could be detected within7nbsp;atherosclerotic plaques and viable specimens of *A. actinomycetemcomitans* and *P. gingivalis* have been isolated from arteriosclerotic tissue (78–80). CVD patients showed a comparatively more extensive and diverse microbial infection of atheromatous plaques in case of comorbidity with PD (81). Also, PD altered the gut microbiota in an atherosclerosis mouse model and in parallel increased intestinal permeability and atherosclerosis severity, suggesting a role for altered gut microbiota in the link between PD and atherosclerosis (82).

### Systemic inflammation

Furthermore, the quality of a systemic inflammatory condition becomes clear when leukocyte numbers and levels of pro-inflammatory markers are determined. Patients with PD displayed increased leukocyte numbers in blood (83) and a large prospective study with a follow-up of 11 years revealed that white blood cell count was longitudinally associated with periodontal disease severity (84). Furthermore, serum concentrations of CRP were elevated in patients affected by PD (85), but also in cases of PD and acute coronary syndrome (86). Moreover, interleukin 6 (IL-6), being a pro-inflammatory cytokine inducing CRP production and mediator of the acute phase response to infection, has been described to increase in case of PD, which might influence comorbidities (87–89). Levels of hs-CRP are used in the clinic to quantify cardiovascular risk that is remaining after intensive lipid-lowering therapy, being referred to as ‘residual inflammatory risk’ (90), and high hs-CRP levels in patients without prevalent CVD were shown to be associated with future adverse vascular events (91). Also, in the CANTOS trial, MI patients that showed high levels of IL-6 despite successful IL-1β blocking suffered from a significant residual inflammatory risk (92), suggesting an important contribution of IL-6 to future cardiovascular events.

Besides CRP, serum amyloid A (SAA) is an important acute phase protein. It supports the cellular inflammatory reaction by its chemotactic function for neutrophils and monocytes, it promotes production of pro-inflammatory cytokines via TLR2 signaling in human gingival fibroblasts and inhibits neutrophil apoptosis (93, 94). Thus, as its involvement in periodontal inflammation has been described (95, 96), increased levels of SAA in serum and gingival crevicular fluid are highly associated with chronic PD (97, 98).

Results from an *in vitro* study on human aortic endothelial cells suggest the SAA-induced expression of endothelial adhesion molecules through TLR2 receptors as a possible mechanism linking PD and atherosclerosis (99). In patients suffering from comorbid CVD and PD, SAA levels have been found to be significantly increased, along with an accumulation of SAA and CRP in samples from carotid atheroma (100). The furthermore noted interactions between SAA and HDL indicate that SAA is involved in lipoprotein regulation, which has been identified as an important element in the pathogenesis of atheromata (101–103).

### Innate immune cell activation

Peripheral blood mononuclear cells from PD patients showed increased expression of pro-inflammatory molecules (such as TNF-α and CCR2), as revealed by single-cell RNAseq (104). Monocytes also demonstrated long-term activation upon exposure to *P. gingivalis*, with a prior microbial infection triggering increased production of IL-6 and TNF-α upon a secondary inflammatory stimulus six days later (83). Furthermore, neutrophils from PD patients showed a hyperreactivity upon stimulation with either LPS or *P. gingivalis*, with increased secretion of IL-8, IL-6, TNF-α and IL-1β (105), increased ROS production in basal conditions (106) as well as upon stimulation of the FcγR (107) and an increased interaction with endothelium (106). Such increased FcγR-triggered ROS production could also be induced upon priming healthy neutrophils with type I interferon (IFN). In this context, PD patients revealed enhanced circulating levels of IFN-α and an increased expression signature of type I IFN-induced genes in peripheral blood neutrophils. Furthermore, periodontitis treatment could reduce IFN-α plasma levels, and FcγR-stimulated neutrophil ROS production to levels observed in controls (108).

PD patients also show an increased frequency of myeloid dendritic cells (myeloid DCs) in blood, and *P. gingivalis* could stimulate monocyte differentiation to immature mDCs *in vitro* (109). Myeloid DCs from patients with chronic PD as well as *P. gingivalis*-infected DCs *in vitro* display an upregulated expression of the homeostatic chemokine receptor CXCR4 but a downregulation of CCR7. Whereas CCR7 confers homing capacity of DCs to secondary lymphoid organs via CCL19, CXCR4-positive cells migrate towards the CXCR4 ligand CXCL12, which was shown to be increasingly expressed by *P. gingivalis*-infected endothelium *in vitro* (110). Also, *P. gingivalis*-infected DCs showed increased expression of the chemokine receptor CCR2 (109), an enhanced inflammatory profile (e.g. with increased expression of C1q, CXCL16 and HSP60/70), a higher secretion of atherosclerotic plaque-destabilizing MMP-9 (109) as well as a downregulated apoptosis and autophagy (111). Combined, this may contribute to enhanced microbial dissemination, atherosclerotic plaque recruitment and inflammatory capacity. Myeloid DCs infected by *P. gingivalis* have also been detected in atherosclerotic lesions within coronary artery biopsies of PD patients with coronary artery disease (109).

### Hyperlipidemia

Patients with PD display increased levels of LDL, oxLDL and triglycerides but reduced levels of HDL, as concluded from a prior meta-analysis study (112). In addition, also structural modifications of both HDL and LDL/VLDL indicative of more pro-inflammatory forms of these lipoprotein particles have been identified in PD patients (113). Post-translational modifications of lipoprotein particles have been increasingly detected in different patient cohorts, including in patients with severe CVD (114) and patients with chronic kidney disease (115). Both in CVD and CKD, these modifications were able to turn LDL into an even more atherogenic lipoprotein particle and modify HDL from a cardioprotective into a detrimental molecule, which contributed to increased cardiovascular risk (114–116).

### Reduced vascular elasticity and endothelial dysfunction

There are different possible parameters to assess cardiovascular health and investigating how those are potentially affected by PD allows to discern further connections, examples being an observed higher systolic blood pressure in cases of tooth loss or an augmented likelihood for hypertension in general (117, 118). Considering the function of blood vessels, an association of PD and dysfunction of the vascular endothelium as well as a sclerotization and increased thickness of the intima and media of the carotid artery has been described (119, 120). Further evidence exists for an increase in pulse wave velocity accompanying PD, indicating a loss in vascular elasticity and thus an increased risk of CVD (121, 122). The regenerative capacity of the endothelium seems to be impaired by the systemic inflammation too: The number of endothelial progenitor cells negatively correlates with clinical parameters of periodontal health like number of teeth, probing depth, bleeding on probing and clinical attachment loss (123). Pathologically elevated serum levels of homocysteine are described as a possible causal cofactor of endothelial damage and atherosclerosis (124, 125). Hyperhomocysteinaemia has been found in cases of PD, adding another possible path of cardiovascular damage linked to periodontitis (126).

### Thrombocyte activation and neutrophil extracellular traps

The expression of CD40-ligand (CD40L) on the surface of thrombocytes is promoted by pathogenic species, especially an infection with *P. gingivalis* is associated with elevated levels of CD40L through TLR 2 and 4 (127). The ligand of CD40 plays a significant role in the development of atherosclerosis and the disintegration of atherosclerotic plaques (128, 129). It enhances haemostasis by stimulating the expression of tissue factor on endothelial cells and macrophages. In addition, increased levels of soluble CD40L associate with myocardial infarction and worsened clinical outcome after acute ischemic stroke (130), making it a potential biomarker for acute cardiovascular events (131).

Additionally, *P. gingivalis* infection promotes thrombocyte aggregation by increasing platelet expression of P-selectin and induces the formation of NETs (132). NETs emerge when neutrophil granulocytes release chromatin and granule proteins and they serve as an innate defense mechanism to catch bacteria, hindering their sprawl and facilitating their exposure to antimicrobial factors (133). Recent findings support a direct interaction of parodontopathogenic species and their products with NETs, possibly evading or neutralizing the antimicrobial potential of the latter. Several species including the potent triad of *P. gingivalis, Tannerella forsythia* and *Treponema denticola* within the red complex as well as *A. actinomycetemcomitans* and *Prevotella intermedia* have been identified to produce DNAses which degrade NETs (134–136). *P. gingivalis*, in particular, is able to citrullinate NET proteins via a peptidylarginine deiminase, neutralizing their positive charge and thereby their pathogen-binding property (137). Despite their primary anti-infective purpose, NETs show possibly detrimental effects too, for instance by promoting activation of factor XII and thrombocytes and formation of atherosclerotic plaques, specifically, if their presence is prolonged and they remain in lesions for an extended period (138, 139). Patients with PD showed increased concentrations of extracellular DNA, nucleosomes and NET-associated myeloperoxidase (MPO) and neutrophil elastase in saliva (140). Moreover, the decomposition of NETs is impaired in patients with untreated PD (141) and, conversely, is amplified after periodontal therapy (142). Nevertheless, detailed information about their specific role in PD and its systemic impact, also in relation to PD-associated CVD, is still missing and requires further investigation.

All combined, these studies reveal an important impact of PD on pathophysiological processes that drive the development and progression of atherosclerotic CVD.

## Animal studies support a contribution of periodontal disease to atherosclerosis

### Combined PD-atherosclerosis model

Given the many confounding factors that can jointly influence PD and atherosclerotic CVD, animal models are essential to demonstrate a causal role of PD to atherosclerosis and to reveal underlying pathophysiological mechanisms. In such animal models, atherosclerosis models are combined with PD induced either by a ligature around the posterior teeth (ligature-induced PD) or by oral gavage with human periodontal pathogens, mostly *P. gingivalis.* Although these models cannot reproduce the natural progression of periodontitis in humans, they enable to investigate the contribution of pathophysiological mediators to pathophysiological mechanisms underlying periodontitis development and/or progression. Overall, PD promoted atherosclerosis in experimental models with mice (80, 143, 144), rabbits (145) as well as pigs (146). In mice, the most applied atherosclerosis models were Apolipoprotein E-deficient (*ApoE^-/-^*) mice on high-fat diet, although also C57BL/6J wild-type mice on long-term high-fat diet as model of metabolic disturbance have been studied.

### Dyslipidemia

Mechanistically, experimental PD enhanced circulatory levels of triglycerides (143, 147, 148), total cholesterol (147, 148) and more specifically VLDL in hyperlipidemic *ApoE^-/-^* mice (143), as well as of oxidized LDL (80, 143). Others observed also a reduction of HDL levels upon infection of mice with *P. gingivalis* (149). However, such effects on HDL or lipid levels in general were not observed in all studies, with for example another study not observing lipid changes in either wild-type or hyperlipidemic *ApoE^-/-^* mice subjected to ligature-induced PD (144), or also HDL remaining unaltered in other studies of experimental PD (147). In relation to PD-induced changes in lipid metabolism (and more specifically increased cholesterol and triglyceride levels), it could be shown that ligature-induced PD in hyperlipidemic *ApoE^-/-^* mice triggered the colonization of the oral cavity and the liver with *F. nucleatum*, which subsequently promoted glycolysis and thereby lipogenesis in hepatocytes (148).

### Vascular inflammation and endothelial dysfunction

Experimental PD induced vascular inflammation with increased expression of IL-6, TNF-α and the endothelial activation marker VCAM-1, as well as with increased activation of the prototypical pro-inflammatory transcription factor NF-κB (150). Experimental PD also triggered endothelial dysfunction in rats, evidenced by a reduced endothelial-dependent vasodilatation (151).

*In vitro*, *P. gingivalis* triggered endothelial inflammation and a dysregulation of the circadian clock genes via TLR-NF-κB signaling, resulting in increased oxidative stress and inflammatory signaling in endothelial cells (152). *P. gingivalis* also induced the shedding of microvesicles by endothelial cells, with a subsequent pro-inflammatory effect towards the endothelium (153). Furthermore, *P. gingivalis* induced endothelial mitochondrial dysfunction, evidenced by an increase in mitochondrial fragmentation and ROS production vs. reduced mitochondrial DNA copy numbers and ATP levels (154). *P. gingivalis* also increased endothelial permeability *in vitro* and in zebrafish larvae by reducing the expression of PECAM1 and VE-Cadherin (76), both important gatekeepers of endothelial cell junctional integrity.

On the other hand, endothelial inflammation in C57BL/6J mice on high-fat diet and infected by *P. gingivalis* was only observed after 28 weeks along with the onset of lipid deposition in aorta and early-stage atherosclerosis, without bacterial infection found in the aortic endothelium itself (155). Instead, *P. gingivalis* did colonize perivascular adipose tissue (PVAT) and triggered PVAT inflammation, increasing the inflammatory milieu with increased Th1 but fewer Treg T-cells, increased levels of pro-inflammatory adipokines (IFNγ, leptin, resistin) but reduced levels of the anti-inflammatory adipokine adiponectin. PVAT inflammation preceded endothelial inflammation and early atherosclerosis in this mouse model, suggesting a potential contribution to atherosclerosis development by increasing systemic inflammation (155).

Treatment with an anti-inflammatory peptide (156) or with rosuvastatin (157) - which not only lowers LDL cholesterol but is also anti-inflammatory - counteracted both PD-induced vascular inflammation and lipid deposition in hyperlipidemic *ApoE^-/-^* mice. Also, topical application of resolvin E1 - a specialized proresolving lipid – prevented PD and reduced systemic inflammation (CRP levels in blood) as well as atherosclerosis upon *P. gingivalis* infection of rabbits on high-cholesterol diet (45).

### Increased systemic inflammation, innate immune activation and trained innate immunity

(Transiently) increased systemic levels of the pro-inflammatory markers CRP, IL-6 and IL-1β have been observed upon ligation-induced PD (144, 151), with stronger effects in hyperlipidemic *ApoE^-/-^* mice compared to wild-type mice on high-fat diet (144). Instead, the atheroprotective factor nitric oxide - which induces vasodilatation and confers endothelial protective properties - was significantly reduced in blood in experimental PD (80, 143).

That experimental PD increased systemic inflammation was also evident from the raised activation status of innate immune cells. PD activated monocytes/macrophages, with increased expression of TNF-α and IL-6 in circulating mononuclear cells of rats subjected to PD (150). In these PD rats, monocytes were also more prone to adhere to endothelium both *in vitro* as *in vivo*, with PD-derived monocytes able to induce pro-inflammatory NF-κB activation in endothelial cells (150). Also, *P. gingivalis* triggered the expression of lectin-like oxidized low-density lipoprotein receptor-1 (LOX-1) in both endothelial cells and monocytes (158), with LOX-1 known to contribute to atherosclerosis by inducing endothelial inflammation and monocyte-endothelial interaction (159). Furthermore, *P. gingivalis* enhanced macrophage foam cell formation by increasing the uptake of oxLDL (160) and blocking lipid efflux (161).

Beyond increased innate immune cell activation, PD was recently shown to trigger inflammation-induced trained myelopoiesis: Ligature-induced PD triggered myelopoiesis in the bone marrow along with transcriptional changes in HSPC in the bone marrow. After resolution of PD and local periodontal inflammation, a secondary systemic challenge with LPS triggered increased myelopoiesis in bone marrow and myeloid cell recruitment to the periphery compared to mice that were previously not subjected to PD (162). Also, *ex vivo*, neutrophils and monocytes from PD-trained mice produced more IL-6 and TNF-α upon a secondary LPS challenge compared to mice that were not previously subjected to PD (162). Mechanistically, long-lasting epigenetic rewiring was identified in HSPC of PD-trained mice (162, 163), with global hypomethylation (163) - generally associated with increased gene transcription - and increased chromatin accessibility of genes suggestive of a biased differentiation towards myeloid cells upon a secondary stimulus as well as an increased pro-inflammatory profile in granulocyte-myeloid progenitor cells (162). Such PD-induced maladaptive training of myelopoiesis was observed along with increased IL-1β levels in the bone marrow and could be shown to be dependent on IL1R-signaling in HSPC. Of note, PD-induced maladaptive innate immune training triggered the susceptibility of the development of other inflammatory comorbidities, as shown for experimental arthritis upon bone marrow transfer of PD-trained bone marrow (162).

### Alterations in adaptive immune cells

Besides effects on myeloid cells, *P. gingivalis* infection increased Th17 cells but downregulated Treg cells in *ApoE^-/-^* mice on high-fat diet, along with increasing atherosclerosis. Whereas Tregs are atheroprotective, Th17 lymphocytes contribute to inflammation and plaque formation by the production of IL-17A (49).

### Altered gut homeostasis

*P. gingivalis*-enhanced atherosclerosis in hyperlipidemic *ApoE^-/-^* mice was associated with changes in the intestinal microflora (82, 164), increased intestinal permeability (82), as well as with raised systemic levels of LPS and TMAO (164) or altered metabolites indicating changes in lipid metabolism and primary bile acid synthesis (82). TMAO is an oxidation product of the gut-derived metabolite TMA, and is associated with higher cardiovascular risk by increasing inflammation (165).

All combined, animal models support a mechanistic contribution of PD to atherosclerosis by driving pathophysiological mechanisms that are known to contribute to the development and progression of atherosclerotic lesions, including endothelial dysfunction, systemic inflammation and innate immune cell activation.

## Beyond atherosclerosis, PD also increases the risk of cardiovascular risk factors as diabetes and chronic kidney disease

Beyond aggravating atherosclerosis, PD also enhances the progression of chronic kidney disease through inflammatory stimuli and oxidative stress (166). Experimental PD led to kidney inflammation with induction of IL-1β and TNF-α, and of tubular and glomerular injury, as shown in *ApoE^-/-^* mice injected with P. gingivalis-LPS (147). Furthermore, the combination of PD and hyperlipidaemia aggravated circulating cholesterol and triglyceride levels as well as kidney inflammation in a synergistic way, with a strong increase in neutrophil infiltration, IL-1β and TNF-α expression in kidney tissue beyond levels reached by each disease alone (147).

Also, experimental PD induced in C57BL/6J mice by infection with the periodontal bacterial species *Aggregatibacter actinomycetemcomitans - a* low-abundance gram-negative periodontal pathobiont - triggered the development of glucose tolerance impairment and insulin resistance along with alterations in the gut microbiota, and - under high-fat diet - induced stronger hepatic steatosis (167). These observations may contribute to the known association of PD and diabetes (168).

However, additional experimental studies are required to pinpoint in more detail the key mediators and mechanisms underlying the association of PD with the aforementioned morbidities.

## Clinical implications & current and future therapy considerations

The effect of PD treatment on cardiovascular health has been assessed in a current S3 guideline (169). In patients without systemic co-morbidities, therapy of PD may reduce the overall cardiometabolic risk and systemic inflammatory biomarkers. For patients affected by a co-morbid NCD, periodontal treatment is suggested for a favorable influence on cardiovascular risk, metabolic control and systemic inflammation based on moderate evidence. However, in case of a co-morbid NCD, it is unclear whether periodontal therapy improves the outcome in severe cases of NCD, as there is no evidence supporting a significant effect on cardiovascular events (169).

As connections in the pathophysiology of CVD and PD have been established, it suggests itself that the therapy of one ailment might prove beneficial for the other. Evidence on the influence of periodontal therapy on vascular function is diverging. An improvement in endothelial function after therapy of PD in patients affected by ST-elevation myocardial infarction has been described (170–173), while other authors found no significant correlation (174, 175). In addition, even after successful therapy the risk for an adverse cardiovascular event remains elevated (176). Periodontal treatment attenuates systemic inflammation, which becomes visible through an improvement of particular inflammatory markers. Accordingly, a lowered concentration of CRP following treatment of PD could be shown (170, 177–182). Blood pressure, IL-6, IL-8 and IFN-γ correlate in a similar way (171, 177, 181, 183). The described amelioration of cardiovascular risk factors by periodontal therapy corroborates the previously suggested correlations between both diseases.

Overall, due to manifold influencing factors, both diseases cannot be completely controlled. On the other hand, the identified disease connections discussed here open various therapeutic ways by targeting inflammation and innate immune activation as link between PD and CVD risk. In these areas, the cardiovascular field is currently investing strong efforts towards clinical translation opportunities. This has been stimulated by the beneficial, lipid-independent effects of IL-1β blockade on cardiovascular outcome in patients with a previous myocardial infarction and high hs-CRP levels in the CANTOS trial (184). These findings boosted an increased focus on inflammation and innate immune activation as targets of therapy in CVD, as recently discussed in detail elsewhere (57, 185). For periodontitis, novel treatments based on the connection between periodontal inflammation, immune-regulatory signaling cascades and the polymicrobial biofilm in the periodontal microenvironment will complement existing anti-infectious therapies and thereby improve clinical outcome (186–188). These current emerging immunomodulatory therapies address inflammation-resolution, relieve inflammation, reduce platelet aggregation and establish a viable local immune microenvironment with subsequent tissue regeneration. Ultimately, this will also be of benefit to address the pathophysiological interaction between PD and atherosclerosis. Overall, unraveling key molecular mediators underlying PD-induced atherosclerosis in further detail may thereby further expand therapeutic options in the future and help to resolve the huge burden of increased cardiovascular risk in PD.

## Conclusions

Taken together, mechanistic and animal studies support a pathophysiological interaction between PD and atherosclerosis beyond sharing of the same risk factors, with PD driving pathophysiological processes that support the development and progression of atherosclerosis. Inflammation and innate immune activation are a central hub in connecting both diseases. Furthermore, they may also represent a key connector of PD and other inflammation-associated diseases such as chronic kidney diseases and diabetes. It is expected that future developments in these fields – as currently highly being investigated in the area of CVD – can also contribute to alleviating comorbid disease presentation. By providing an overview of the current understanding of PD, atherosclerosis and their crosstalk, this review aims to support future research efforts that can uncover novel mechanistic insights into comorbidity development in PD as important step towards advancing clinical translation opportunities.
